# A report on the international conference on environmental mutagenesis in relation to human health held during the annual meeting of the Environmental Mutagen Society of India, January 29–31, 2026

**DOI:** 10.1186/s41021-026-00359-x

**Published:** 2026-05-14

**Authors:** Bani Bandana Ganguly

**Affiliations:** 1 MGM Centre for Genetics Research and Diagnosis, MGM New Bombay Hospital, Vashi Sector 3, Navi Mumbai, 400703 India; 2 MGM Institute of Health Sciences, Navi Mumbai, 410209 India

**Keywords:** Environmental Mutagen Society of India (EMSI), 48th EMSI conference, Environmental mutagenesis, Phyto-remediation of genotoxicity

## Abstract

The 48th Annual Meeting and International Conference of the Environmental Mutagen Society of India (EMSI) on ‘Environmental Mutagenesis & Epigenomics in Relation to Human Health’ was held at Jamshedpur Co-Operative College, in association with Kolhan University, Jharkhand, India, from January 29–31, 2026. There were 141 deliberations in total, with participation from researchers, academicians, Vice-Chancellors, and state government officials from India and eight other countries. The scientific topics, including environmental impact on humans and aquaculture, transgenerational plant protection, molecular insights into cancer research, plants with antimutagenic potential, and sustainable agriculture through the use of bio-pesticides and bio-fertilizers, broadly justified the conference theme. The molecular mechanisms of pathogenesis were discussed through lectures on signalling pathways, gene expression, and DNA damage and repair, highlighting targeted drug development. Additionally, in silico docking of synthetic drugs and nanoparticles was discussed in detail. Notably, nanotoxicology, microplastics, airborne particulate matter, and prenatal arsenic exposure were shown to have a significant impact on human health. As Jharkhand and neighbouring states depend largely on agricultural yield, discussions on the use of plant-based medicines, harnessing infection and immunity, and agricultural eco-toxicology suggested ways to protect farmers’ health and the food chain from the overuse of chemicals. Altogether, the deliberations supported several Sustainable Development Goals and highlighted cost-effective agricultural modalities. These messages were disseminated to the public through local media via daily briefings. Notably, this EMSI conference provided a platform for scientific exchange that attracted administrators and pollution control regulators aimed at protecting human health by mitigating environmental exposure.

## Opening of the conference

**Fig. 1:**
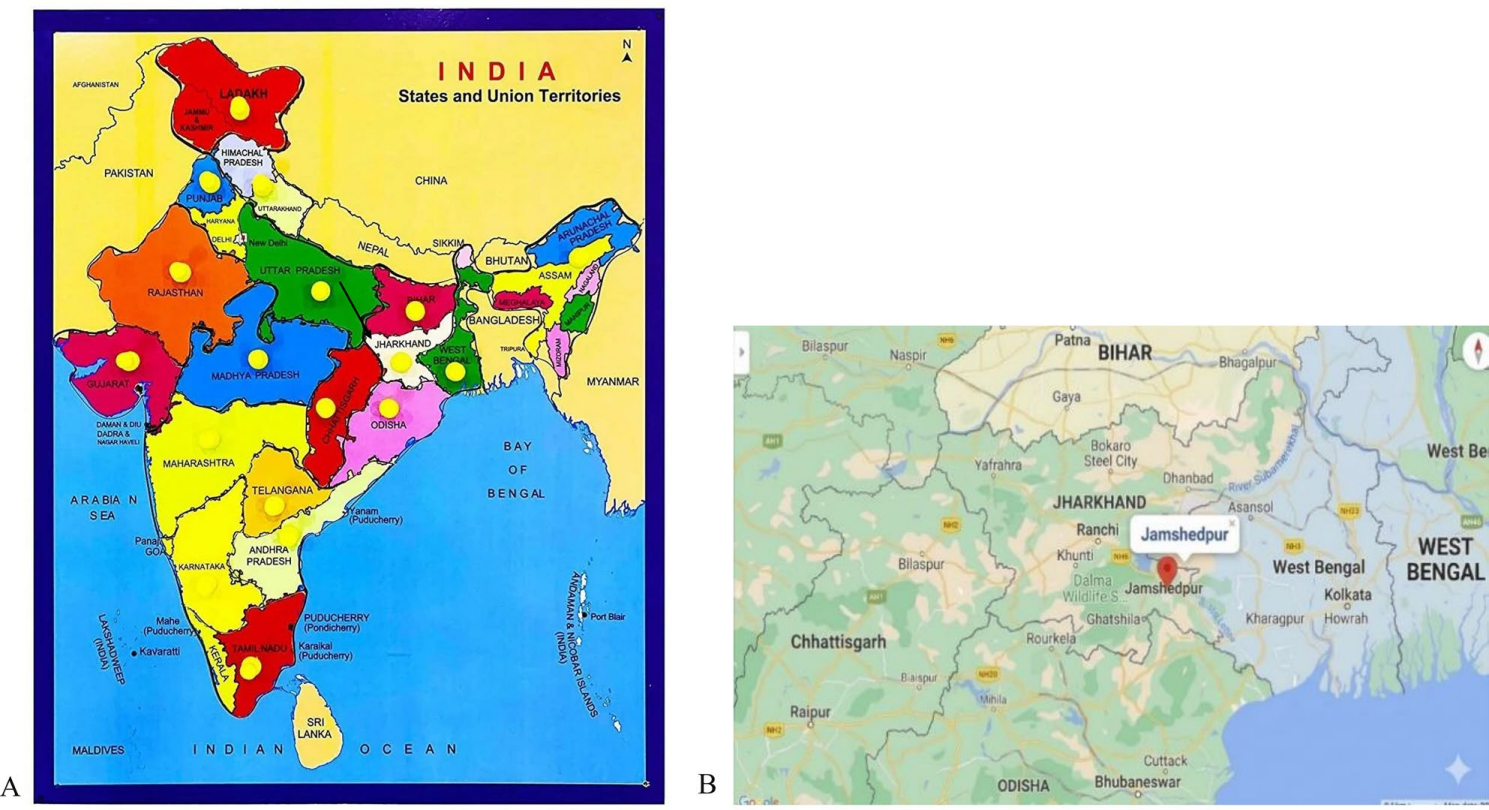
The map shows the location of Jamshedpur, Jharkhand, and adjoining states: **A**. In the whole of India, **B**. Location of Jamshedpur and other important cities in Jharkhand

The 48th Annual Meeting and International Conference of the Environmental Mutagen Society of India (EMSI) on ‘Environmental Mutagenesis & Epigenomics in Relation to Human Health’ was held at the Jamshedpur Co-Operative College, Jamshedpur, in association with Kolhan University, Chaibasa, Jharkhand, India from January 29–31, 2026. The meeting was organised by Dr. Amar Singh, Dr. Vishnu Shankar Sinha, Dr. Ram Prasad, Dr. Brajesh Kumar, and Dr. Neeta Gupta, who served as Chairperson, Organizing Secretary, Program Director, Convenor and Joint Convenor, respectively. The meeting was opened by the Committee in presence of the Hon’ble Governor of the State, the President and Secretary of EMSI, the Vice Chancellor of Kolhan University and many other dignitaries from Higher Education and other Government Departments, following the Indian cultural protocol of lamp lighting. The Governor, who is also the Chancellor of State-Government Universities in Jharkhand, was welcomed by a vibrant tribal folk dance performed by the students and faculties of Jamshedpur Co-operative College. The opening lectures delivered by those key persons were inspiring as they covered the key aspects of environmental mutagenesis, carcinogenesis, drug development, and most importantly, control of the environmental contamination and associated regulatory measures. The conference venue, Jamshedpur, is known as the ‘Steel City’ of India and is located in the state of Jharkhand in Eastern India (Fig. 1). The weather was pleasant during the conference [1]. Jharkhand covers 79,716 km^2^ and has a population of 32,988,134 (as of November 2000; source: https://www.mapsofindia.com/maps/india/india-political-map.htm, accessed April 9, 2026). Ranchi is the capital of the state, while Dhanbad, Bokaro, Mango, and Jamshedpur are other prominent industrial cities.

EMSI was established in 1975 by a pioneering group of scientists, primarily from the Bio-science Group of Bhabha Atomic Research Centre in Mumbai, India. The mission of the society is to promote education and research in the field of mutagenesis and to provide a scientific forum for those engaged in or interested in the application of knowledge regarding the mechanisms of mutagenesis. Through its annual conferences, EMSI aims to disseminate information on the association of mutagenicity with carcinogenicity and teratogenicity, testing methodology for evaluating the mutagenic potential of chemicals and drugs, and the correlation of chemical structure with mutagenicity. Its annual meeting is typically held in conjunction with a conference hosted by institutions across the country. The conference platform facilitates the exchange of scientific ideas and information regarding mutagenesis at both experimental and clinical levels. EMSI is affiliated with the Asian Association of Environmental Mutagen Societies (AAEMS) and the International Association of Environmental Mutagenesis and Genomics Societies (IAEMGS).

## Content of the conference

The conference featured six plenary (PL) and forty two invited lectures (IL), along with forty six oral (OP) and forty seven poster presentations (PP) covering diverse aspects of environmental impacts on living systems. The participation of junior and senior researchers, academicians, regulators, Chancellors and Vice-Chancellors, and other Government Officials from nearly all undergraduate, postgraduate, and research institutions was notable. This likely marked first time in EMSI history that such broad participation occurred. The conference theme, ‘Current Perspectives on Environmental Mutagenesis and Epigenomics in Relation to Human Health’, included several sub-themes. The topics broadly covered: toxicology, mutagenesis, carcinogenesis, nanotoxicology, and epigenomics; occupational and environmental exposure to industrial and natural hazards; gene-environment interactions, including DNA damage and repair mechanisms, signalling pathways, and oxidative stress; impacts on human health and epidemiology; herbal pesticides and drugs; and regulatory policies pertaining to control and management of environmental hazards. Mutagenesis stimulates a genome-wide biological response after exposure to environmental toxicants and stressors, and gene-environment interactions [2, 3].

**Fig. 2:**
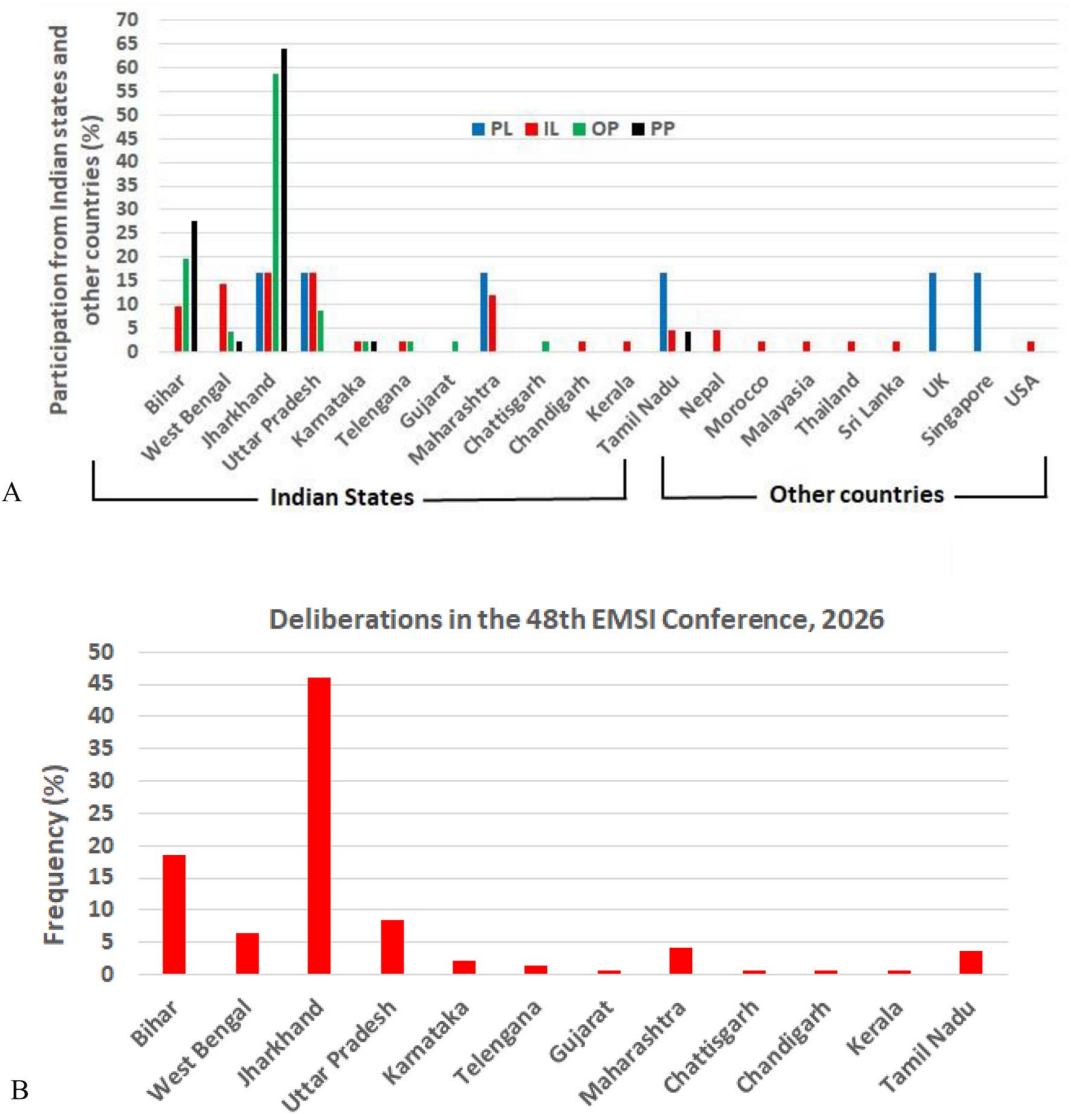
Participation and deliberations in the conference: **A**. Presentation-wise participation from different Indian states and other countries (frequencies are calculated on individual group-total) (Pl, plenary lecture; IL, invited lecture; OP, oral presentation; PP, poster presentation); **B**. Overall participation in the conference

EMSI organizes an annual conference in different parts of the country to facilitate wider awareness and participation. However, participation is generally skewed toward the organizing state and the neighbouring ones. Thus, the nation-wide participation in this conference was dispersed rather than uniform (Fig. 2A, B). International participation was limited, consisting of a single presentations from eight countries (including two from Nepal), likely due to scheduling conflicts with other conferences and potential attendance at ICEM 2026. Nevertheless, the presentation of Bani Bandana Ganguly of the MGM Institute of Health Sciences (Mumbai, India) provided a comprehensive overview of the Indian context of genotoxicity research, including the institutions, departments, and scientists involved, as well as their specific research niches and technological applications [1]. She noted that while several registered professional societies in India conduct toxicological research and organize conferences involving national and international experts, a serious gap persists: these societies neither have exchange information among themselves nor maintain a system for capturing the overall scale or outcomes of genotoxicity research. While reviewing the field, technological advancements, and institutions or universities involved in genotoxicity research, the authors found it a difficult task to collect comprehensive data on the aspect using Google search, PubMed, Scopus and other databases. The data remained incomplete, as many core researchers of different institutions and their research outcome were missed if their research keywords did not align with the search term (Ganguly et al. unpublished). The outcome of her search suggested that instead of having many societies with similar objectives in a country, a single one can include the complete information on all aspects of mutagenesis, carcinogenesis, and toxico-genomics research. Such deficiency and society’s mission can be fulfilled through a cohesive involvement of the executive committee members or working committee of the societies for yielding a better outcome. The societies shall also highlight the upcoming conferences and workshops in the field of genotoxicity research in their website for attracting global participants and facilitating technological exchanges.

### Impact on human health

The majority of the deliberations focused on the environmental impact on human health. The first plenary lecture, delivered by Professor Awadesh Jha of Plymouth University (United Kingdom), discussed the ecotoxicological and epigenetic impacts of tritium, a radionuclide of significant environmental and human health concern. Tritium (^3^H) is a growing global concern due to its massive production in fission reactors and its critical role in future fusion technologies. Unlike many other radionuclides, tritium’s high mobility and presence in water (as tritiated water or HTO) allow it to integrate easily into biological systems and the human food chain. The key insights included the genotoxic and epigenetic impacts observed via significant DNA methylation in marine organisms such as mussels. Tritium’s tissue-specific bioaccumulation indicated heavy accumulation in the gut and gills of marine bivalves, suggesting uneven distribution [4]. Furthermore, DNA methylation in the gut was negatively correlated with tritium concentration in DNA. The study highlighted an urgent need to investigate trans-generational and bystander effects, especially as future fusion energy could increase environmental releases by orders of magnitude compared to current reactors. Finally, the lecture addressed the link of ecological and human health regarding radioactive waste leaking into ecosystems, noting that 95% of human cancers are associated with environmental exposure to toxicants.

Exposure to microplastics can cause a variety of diseases as they accumulate in the food chain and drinking water, subsequently releasing toxic chemicals. These chemicals can induce inflammatory responses, oxidative stress, and cell death in the kidneys, ultimately leading to renal fibrosis. Transcriptome analysis has revealed that chronic microplastic exposure can alter expression of multiple genes related to immune response and circadian rhythms [5]. Vikas Srivastava of Indian Institute of Toxicology Research (IITR) (Lucknow) discussed the effects of environmental microplastics on renal fibrosis and their synergism with arsenic, highlighting the exposure-risks on chronic kidney disease. Additionally, studies on prenatal arsenic exposure revealed promotion of adipocyte dysfunction and insulin resistance via epigenetic modulation of TNF-α expression in mice, contributing to the early onset of metabolic syndrome in the offspring [6]. The study further demonstrated that prenatal arsenic exposure can induce KIP/NF-kB/ERK1/2-mediated early-onset kidney disease in mouse offspring [7]. Environmental factors contributing to health hazards and prevalence of the ZIKA virus were discussed in the context of current global scenario by T. Mariappan of ICMR-Vector Control Research Centre (Madurai). Similarly, Gaurav Sharma of Post Graduate Institute of Medical Education & Research (Chandigarh) presented on the diverse, heterogenous distribution of HLA-alleles and their phylogenetic relatedness. This research facilitates plausible donor selection for stem cell transplantation in various hematopoietic conditions [8].

### Plant pathogens and sustainable agriculture

Food security and safety have become increasingly challenging in the face of population growth, increasing environmental contaminants and alarming rate of pesticide use. Studies on phyto-components as alternatives to synthetic or chemical products are vital for controlling environmental pollution and contribution to sustainable developmental goals. Interestingly, plants possess an innate immune system that identifies the conserved cell surface molecules of most pathogens to prevent infection; however, this adaptation can significantly reduce agricultural growth and yield. Alongside controlling pre-programmed defense reactions against infections, these systems also stimulates responses to selected environmental stress signals, a mechanism defined as defence priming. Discussion on defense priming in plant growth-promoting rhizobacteria (PGPR) by Prashant Singh of Banaras Hindu University (BHU; Banaras, India), was particularly significant. Notably, PGPR has emerged as a powerful and sustainable approach to fortifying plant defense mechanisms. The presentation highlighted the mechanisms of PGPR-mediated priming, focusing on its ability to moderate plant physiology, increase metabolite production and antioxidant enzyme activity, and improve overall tolerance to biotic and abiotic stresses. By inducing a pre-conditioned state of alertness, PGPR enables plants to mount faster and more robust defenses against environmental challenges. This approach enhances resilience to pathogens and environmental stress while supporting sustainable, low-pesticide farming [9]. Furthermore, the insights into this technique demonstrated its significance as a sustainable strategy for protecting crops with minimal environmental impact. The lecture also noted that certain microbes surrounding crop plants are beneficial, as they enhance growth and provide protection against numerous diseases. Despite the lack of full protection from defence priming, its broad-spectrum durability and trans-generational inheritance make it attractive for integrated disease management.

Additionally, Debashish Dey and his team of BHU discussed the broad-spectrum defense activation of the Harpin gene (HrpZ2), derived from *Pseudomonas syringae* strain MTCC-11,950, in host and non-host plants. Infiltration of the harpin protein induced a hypersensitive response by increasing the activity of defense-related enzymes, such as polyphenol oxidase and phenylalanine ammonia-lyase. These findings indicate its potential as an immune booster and bio-pesticide, suggesting an eco-friendly alternative to chemical pesticides for disease resistance management and sustainable agricultural yield [10].

Fungal secondary metabolites, produced during different phases of their growth, have gained significant attention over the last decade due to their potential in biological and environmental bioremediation. Their use in the formulation of novel pharmaceuticals and biopesticides is increasing across the healthcare, environmental clean-up, and biotechnology industries. Furthermore, these metabolites have wide applications in dairy, beverage, and food industries due to their fermentation and preservation efficacies [11]. The utility of these metabolites in sustainable agriculture was discussed by Ravi Kumar Singh of Magadh University (Bihar, India), who emphasized their role in controlling plant infections and increasing agricultural yields across diverse environmental conditions.

Along this lines, Ram Prasad of Mahatma Gandhi Central University (Motihari, Bihar) explained the role of endophytic fungi in plant growth promotion (e.g., Indole Acetic Acid, siderophores), sustainable agriculture, and their association with medicinal plants and crops. The endophytic fungi studied have shown potential to improve plant health by increasing tolerance to abiotic stresses. These fungi are recognized as an eco-friendly and safe alternatives to chemical fertilizers and pesticides, aimed at enhancing productivity while reducing agricultural waste. Studies have indicated that root endophytic fungi are associated with various plants; specifically, their interaction with microorganisms like *Piriformospora indica* can boost productivity. His research also highlighted that endophytes can produce valuable bioactive compounds that can be harnessed for agricultural and pharmaceutical purposes [12]. Additionally, research of his team also explored the use of green-synthesized nanofertilizers and microorganisms to support plant health, nutrient absorption, and overall productivity [13]. Green synthesized nanofertilizers and plant growth promoters offer new hope for sustainable development goals, potentially revolutionizing agricultural practices and contributing to environmental sustainability.

Several lectures on the reduction of chemicals in agriculture addressed concerns regarding crop-environment interactions and their implications to human health via the food chain. While the Green Revolution and agricultural sustainability efforts have increased crop yields, the undue application of chemical fertilizers and pesticides has caused significant deleterious effects on the agro-ecosystem and human health, particularly among farmers. Besides fungal bio-protection, plant-based natural compounds are emerging as sustainable alternatives to chemical pesticides. The development and use of bio-pesticides could reduce the burden of environmental contamination and hazardous risk to the biosphere while preventing insect resistance. Investigations into the isolation of chemical components from medicinal plants, well known to the local tribal communities of Jharkhand and Bihar, were discussed by Vibha Pandey of Nilamber-Pitamber University, Jharkhand [14]. Anand Kishor of V. K. S. University (Ara, Bihar) discussed the use of vermicompost and its nutritional benefits to plants as well as its effects on the chemical constituents of different plant parts. This system can provide a sustainable and eco-friendly solution for enhancing production and pest management in grain crops. Vermicompost not only improves nutrient availability to plants but also prevents soil pollution and protects soil microorganisms. Notably, vermicomposting was highlighted as an eco-friendly and cost-effective approach for sustainable agriculture practices.

Satyakam Patnaik of IITR (Lucknow, India), presented the promising pest control effects of nano-agrochemicals, which could lead to a paradigm shift in sustainable crop production. He highlighted the benefits of agro-nanotechnology for controlled-release formulations (CRF), addressing the issue of pesticide overuse common in conventional pest control practices [15]. These advancements support sustainable yields without compromising the farmers’ health or contributing to adverse environmental impacts. Additionally, the effects of priming (hydro-/halo-) with K- and Mg-salts on germination and growth of rice plants were also discussed.

### Environmental exposures, xenobiotics and biomolecules

The environment consists of a complex mixture of radiation, gases, chemicals, and nano-chemicals originating from both natural sources and human activity. These compounds possess significant mutagenic and carcinogenic potential, impacting both the ecosphere and human health. Silica nanoparticles (SiO2) are abundant in the environment due to their diverse applications in food, drug delivery, and construction. Aruna Satish of IITR (Lucknow), discussed the multigenerational impact of SiO2 toxicity on the model organism *Caenorhabditis elegans*. Her research showed that downregulation of transcription factor (daf-2) and vitellogenin (vit-2 and vit-6) expression was accompanied by increased oxidative stress and germline apoptosis [16]. Monitoring across 11 generations revealed a decline in progeny count. Notably, after six generations of exposure, it took five generations of recovery of the worms to regain their original vitality. The study suggests a cumulative effect of SiO2 on reproductive outcomes across generations, which could ultimately disrupt the ecological balance.

Environmental arsenic exposure is a pandemic issue in India. Its adverse health effects include cognitive impairment in experimental animal models and children living in the arsenic-affected areas. Debabrata Ghosh of IITR (Lucknow), presented findings on the effects of gestational exposure to sodium arsenite, which resulted in elevated microglial proliferation, reactive oxygen species (ROS), nitric oxide (NO), proinflammatory cytokines and chemokines in BALB/c mice. This exposure promoted phagocytosis via the phagocytic receptor TREM2, while repressing the expression of synaptic proteins SNAP-25 and PSD-95 [17]. Collectively, this enhanced neuronal pruning led to impairments in learning and memory function.

Ravi Ram Kristipati of the same institute (IITR) discussed the link between xenobiotic exposure during development and incidence of type 2 diabetes (T2D) using *Drosophila melanogaster* model. The research highlighted that exposure to atrazine, a common herbicide, triggered oxidative stress-mediated c-Jun N-terminal kinase (JNK) signalling, resulting in insulin resistance. In contrast, exposure to dichlorvos (DDVP), an organophosphorus pesticide, activated caspase-mediated cell death pathways [18]. The study demonstrated that developmental exposure to DDVP leads to insulin deficiency, characteristic of type 1 diabetes (T1D), while atrazine exposure induces hallmarks of T2D. These findings suggest that pesticide-mediated diabetogenesis is a critical risk factor during organismal development; the researchers recommend the use of anti-oxidants to mitigate the oxidative stress caused by the diabetogenic chemicals. In this direction, research by Ahmed Ali of Mumbai University (Mumbai, India), demonstrated glycation-induced structural alterations of DNA and proteins, as well as the efficacy of natural products on antidiabetic and antiglycation treatments [19].

In vitro studies on nano-particles (NP) highlighted the genotoxicity of silver-NP and epigenetic study of trans-generational toxicity of silica-NPs. The genotoxic and anticancer effects of silver nanoparticles was discussed by Prakash Hande of National University of Singapore. His research revealed chromosomal aberrations, mitochondrial toxicity, and inhibition of cell proliferation in a dose-dependent manner [20]. Similarly, the antimicrobial, anti-inflammatory, and dental applications of eugenol nanoparticles were discussed by Fatima Zahra Kamal of Higher Institute of Nursing Professions and Health Techniques (ISPITS), Casablanca, Morocco [21].

The genotoxic potential of environmental particulate matter (PM) and its mechanisms of action were demonstrated in relation to human health. A transcriptomic and molecular study of ambient PM_2.5_ exposure risk, specifically involving EGFR/PI3K/AKT/mTOR and JAK/STAT signalling, on lung cancer in vivo in rural and urban populations was presented by Dona Sinha’s group at Chittaranjan National Cancer Institute (CNCI) (Kolkata, India) [22]. Additionally, an in vitro study of PM_2.5/0.1_ by Alok Pandey’s team at IITR (Lucknow) detailed the mechanisms of mitochondrial dysfunction, oxidative stress, NF-κB/p38 MAPK signalling, and genotoxicity, highlighting increased expression of γ-H2AX and DNA repair proteins (OGG-1/APE-1/PARP-1) alongside irreparable DNA double strand breaks [23]. Kumari Swarnim of Ranchi Women’s College (Ranchi, India) discussed the potential mechanisms of pesticide-related mutagenicity. Her studies showed that 50 out of 228 tested pesticides, specifically organophosphates, halogenated alkanes, and dithiocarbamates, were mutagenic in bacterial reversion-assay systems (Ames test) [1].

Vinay Jain of Bhabha Atomic Research Centre (BARC) (Mumbai, India) presented an evaluation of the mutational landscape in humans living in high natural background radiation areas in coastal Kerala. The findings indicated no detectable genetic harm, suggesting that adaptive molecular mechanisms may maintain genome stability. Another researcher from the same group assessed human health risks in response to ionizing radiation. She discussed the current perspectives in radiation protection science, highlighting that radiation-exposure may lead to adaptation and subsequent alterations in gene expression [24]. Additionally, Devashish Rath, also from BARC, presented insights into the biodegradation of di- and tributyl phosphate by metallo-phosphoesterase extracted from *Sphingobium sp.* RSMS microorganism.

### Plants with antimugenic potential

Plants are essential natural resources possessing antimutagenic, antioxidant, and anticarcinogenic properties, while serving as the foundational component of the food chain. Several lectures addressed the conference theme by covering topics such as: manufacturing of plant-based bio-plastics; antihypertensive and hypotensive management with ayurvedic preparations; enhancement of antibiotic efficacy via plants extracts; and the use of phyto-active compounds (e.g., *Annona muricata*,* Oroxylum indicum;* and *Rheum emodii*) to induce cell death in breast and dermal cancer cell lines [1]. Additionally, modulating effects of various plant extracts against adriamycin-induced mutagenesis and genotoxicity were demonstrated, primarily using mouse models (germ cells and bone marrow erythrocytes). Results indicated that combining *Curcuma* rhizome extract (CRE) and *Piper* fruit extract (PFE) significantly reduced the percentage of abnormal sperm induced by adriamycin, suggesting their potential as safe antimutagenic agents in chemotherapeutic strategies [25]. Furthermore, *Boesenbergia rotunda* (L.), commonly known as fingerroot, was highlighted as a medicinal plant with significant potential for dietary supplements development. Its major flavonoids, pinostrobin and pinocembrin, display osteogenic functions alongside hyperglycemia control. Development of a dietary supplement in tablet form containing these flavonoids for prevention of osteoporosis in postmenopausal women was presented by Nuttinee Teerakulkittipong of Burapha University (Chonburi, Thailand) [1]. The deliberation on conservation of gene pool of medicinal plants further reinforced the conference theme.

### Impact on fisheries

The environmental impact on fisheries and aquaculture was emphasized through several key topics. Discussions on the role of diverse freshwater habitats highlighted their importance to global livelihoods of fisheries and the food chain. Microplastic contamination in aquaculture remains a global crisis, impacting human health through seafood consumption. Deep Narayan Shah of Tribhuvan University (Kathmandu, Nepal) presented on the scale of microplastic contamination and its impact on freshwater biota in Seti and Bagmati River Basins of Nepal [26]. Notably, the hilly terrains of Nepal contain more than 6,000 rivers where watersheds of varying altitudes (60 m to 8848 m) support 252 fish species. Various remedial and management measures were discussed, including: nanobubble aeration to mitigate geosmin and eutrophication; optimization of C/N ratios, heterotrophic bacteria, and plankton abundance for different fish varieties; and the impact of seasonal water quality variations on fish diversity. Additionally, the use of papaya seeds to reduce reproductive performance of Nile Tilapia (*Oreochromis niloticus*) was explored, leading to a suggested “pond education programme for adolescent students” of Chitwan and Nawalparasi districts of Nepal [27, 28]. This issue of microplastic effect on aquaculture was further addressed by researchers of the Central University of Kerala and other Indian institutions. Another serious concern involves the degradation of pond ecosystems by urban run-off, household sewage, and ritualistic activities, which have led to an increased incidence of pathogenic bacteria and antimicrobial resistance across a broad spectrum of pathogens.

Subha Bhassu of the University of Malaya (Malaysia), explained the spatial epigenomics of pollutant and pathogen exposure, alongside their cellular mechanisms and human health implications. Their epigenomic and gut-metagenomic study of shrimps, as well as the use of CRISPR/Cas9 editing to inhibit fungal sporulation; and so on indicate a diverse research horizon aimed at the betterment of human life. Her team is deeply involved in dissecting environmental DNA (eDNA) modulation in aquatic organisms and its subsequent impact on human health [29]. Similarly, Poorna C. Piyathilaka of the University of Colombo (Sri Lanka) provided molecular insights into environmental and probiotic bacteria in relation to human health. Her research focuses on the molecular assessment of probiotic bacteria used in local aquaculture to detect potential risks, specifically analyzing antibiotic resistance within these probiotics. She highlighted the risks associated with tetracycline-class antibiotics in aquaculture environments and continues to investigate microbial water quality and pathogen screening. Testing of antibiotic resistance genes (tetA/B, qnrA/B/S) in environmental aquatic bacterial isolates and commercially used probiotics against tetracycline and ciprofloxacin indicated environmental persistence and horizontal gene transfer [1].

### Molecular insights into cancer research

Molecular insights into cancer and other disease patho-mechanisms directed the discussion toward signalling pathways and genomic/epigenomic-based therapeutic targets. Researchers from CNCI (Kolkata, India) presented several key findings, including: a flavonyl-thiazolinedione hybrid-based organo-selenocyanate small molecule that acts as a chemo-protectant against cyclophosphamide-induced toxicity while maintaining sensitivity toward breast cancer cells tested in mice; a synthetic approach to drug delivery that conjugates DNA-targeting moieties with clinically approved non-oncologic drugs; and the stage-specific increase in glutamine metabolic markers of cisplatin therapy in cervical cancer. These studies highlighted the molecular basis of disease-development and potential control strategies [30, 31]. Along these lines, PK Suresh of Vellore Institute of Technology (Vellore, India) discussed in vitro and in silico approaches for anti-cancer drug development, focussing on the cell death-induction potential of natural molecules, extracts, and repurposed drugs [32]. Kaustav Sarkar of SRM Institute of Science and Technology (Chennai, India) demonstrated epigenetic and epi-transcriptomic modifications in T-helper cells within the context of coronary artery disease and cancer. His work analysed TP53 and BRCA1 expression, alongside histone trimethylation at lysine residue 27 (K27) and DNA/RNA methylation [33]. Furthermore, Sathees Raghavan of Indian Institute of Science (Bangalore, India) provided novel insights into DNA double-strand break repair mechanisms by redefining of non-homologous end joining (NHEJ). His research explained how the interaction of BCL2 with Ku70, a core NHEJ protein, downregulates repair in cancer cells. These findings emphasize the potential of NHEJ inhibitors and the need for characterizing tissue-specific NHEJ mechanisms for cancer therapeutics [34].

### Communication with commoners

Besides environmental pollution, there were deliberations on how natural hazards such as volcano, flood, earthquake, etc. pose a significant traumatic impact on human psychology through loss of economy in various forms. The areas highlighted the unexplored stress-related consequences on human health. Such integrated approach may provide a holistic One-Health framework for disease surveillance aiming with a direct relevance to human health. Above all, daily news-coverage on the scientific deliberations and one-on-one interaction with the scientists on region-specific prevalent disease-types in local language (Hindi) gained attraction of the common people of different levels of understanding. Alongside, active participation of the Ministry of Higher and Technical Education Department of the Jharkhand State considered the scientific topics and discussions held during the conference with a serious note for the improvement of education, research and public health services in the States of Jharkhand and adjoining state - Bihar.

## Cultural refreshment

**Fig. 3:**
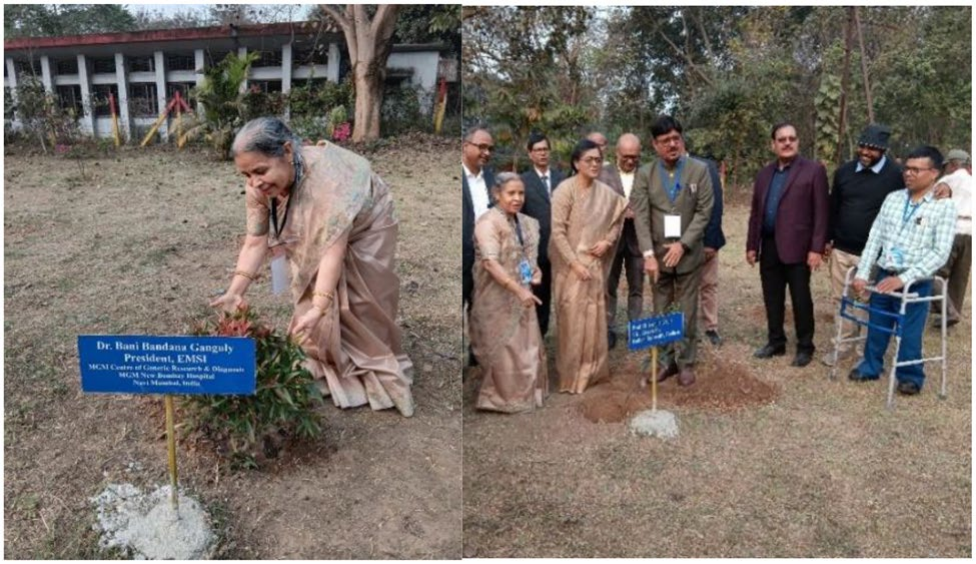
Tree plantation organized during the 48th EMSI conference held at Jamshedpur

The societal aspect of the conference was quite appreciable. The cultural programme in the evening of the first two days was unforgettable, which was so complete with music, poetries, and dance activities. The local tribal dance and ‘Chou’ dance of Purulia were so rhythmic and powerful, which dictated the essence of cultural legacy in the present generation. Most importantly, most of the performers belong to the organizing institute. Also, tree plantation by some of the key participants in the venue-campus was a memorable event (Fig. 3). Notably, the organizer made a very helpful and comfortable arrangements on accommodation and local logistics from Ranchi airport, the nearest one. Daily multi-cuisine arrangements for the multi-cultural nation-India, right from breakfast till dinner, was truly noteworthy, and that was nutritionally so balanced with veg-/non-veg items covering continental/inter-continental menus.

## EMSI committee meetings

**Fig. 4:**
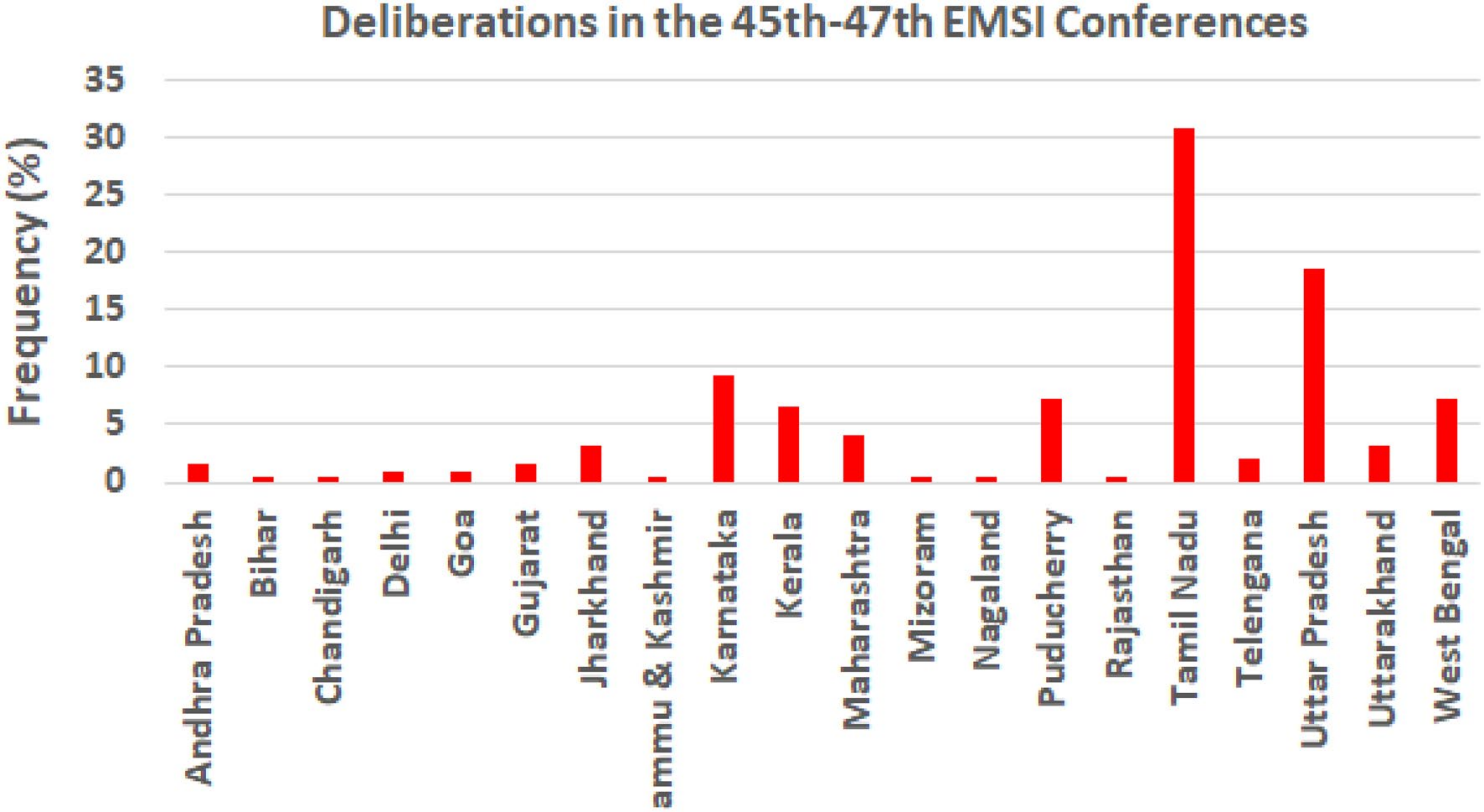
Region-wise distribution of participation in the past three conferences (45th-47th). Note: The figures have been collected from abstract books of the 45th to 47th EMSI conferences, which were held in Aligarh Muslim University, Uttar Pradesh; Central University of Kerala, Kerala; and Annamalai University, Tamil Nadu, respectively [35–37]

EMSI conducted meetings with the members of the Executive Committee (EC) and a General Body (GB) meeting with all members during the first two days. The meeting declared the result of the election and the composition of the new EC, and financial status of the society. Discussion also included modalities to increase in conference registration and EMSI members, and selection of the venue of the 49th EMSI conference. Overall, the participation in the EMSI conferences has become almost regionalized (Fig. 4) [2–4].

## Conclusion

The conference ended with a valedictory session on January 31, 2026, attended by the executives of the Higher Education Departments and several past and present Vice-Chancellors from Jharkhand and Bihar. The session reviewed the event’s achievements and expressed a strong commitment to organizing similar future forums. A unique highlight was the collective pledge taken to control environmental issues and promote awareness of their impact on living organisms.

The 48th Annual Meeting and International Conference of the Environmental Mutagen Society of India (EMSI) concluded by fulfilling its central theme through diverse and high-impact scientific deliberations. The sessions demonstrated how environmental stressors, ranging from xenobiotics to ionizing radiation, impact human health, fisheries, and agricultural ecosystems. Presentations highlighted signalling pathways, oxidative stress-related DNA damage, and error-prone repair mechanisms, offering molecular insights into cancer development and potential therapeutic targets. Researchers addressed the molecular basis of disease onset, including transgenerational effects of nanoparticles and the genotoxic potential of common pesticides. Significant focus was placed on plants with antimutagenic potential and the use of phyto-active compounds to mitigate toxicity and induce cell death in cancer cell lines. For regions like Jharkhand and its neighbours that are heavily dependent on agriculture, the conference provided critical strategies for chemical reduction by implementing plant-based pesticides, bio-fertilizers, and infection-harnessing techniques to protect the food chain and farmers’ health from chemical overuse. Sustainable practices emphasized use of cost-effective agricultural modalities that align with global Sustainable Development Goals. Collectively, this conference served as a vital platform for public awareness and regulatory bridge through involvement of administrators and pollution control regulators, aiming to mitigate environmental exposure and protect public health. These critical messages were further disseminated to the general public through daily media briefings, ensuring the scientific outcomes reached a broader audience.

## Data Availability

No datasets were generated or analysed during the current study.

## Declarations

No AI was used for generating this report, but used only for grammatical corrections.
